# Physically Violent Parental Practices: A Cross-Cultural Study in Cameroon, Switzerland, and Togo

**DOI:** 10.1007/s40653-023-00564-8

**Published:** 2023-07-08

**Authors:** Caroline Naudin, Veronica Gatti, Kossi B. Kounou, Claude-Olivier Bagnéken, Marie-Chantale Ntjam, Marie-Ève Clément, Fabrice Brodard

**Affiliations:** 1 Family and Development Research Center (FADO), Institute of Psychology, University of Lausanne, CH-1015 Lausanne, Switzerland; 2 Research Team in Psychology, Orientation and Development, University of Lomé, Lomé, Togo; 3 Association EMIDA, Yaoundé, Cameroon; 4 Department of Psychology, University of Douala, Douala, Cameroon; 5 Department of Psychoeducation and Psychology, University of Québec in Outaouais, Saint-Jérome, Canada

**Keywords:** Adverse childhood experiences, Attitudes, Cultural differences, Parenting, Physical violence, Sub-Saharan Africa

## Abstract

Violence against children and adolescents is a widespread problem. However, most studies conducted in this field has been carried out in Western countries and studies are needed in non-Western countries, especially in Sub-Saharan Africa, where rates of child physical violence are high. The present study aimed firstly to document the different forms of physical violence and attitudes toward corporal punishment (CP) across Cameroon, Switzerland, and Togo. The second objective aimed, on the one hand, to understand the influence of cultural context, childhood physical abuse, and parental attitudes on physically violent parental practices in these three different cultural contexts. On the other, this study aimed to investigate the mediating role of childhood physical abuse and parental attitudes on the effect of cultural contexts on parental practices. Five hundred and forty-seven parents from Togo, Cameroon, and Switzerland filled out questionnaires concerning violent parental practices (ICAST-P), childhood physical abuse (CTQ-SF), and parental attitudes in favor of CP. Firstly, results highlighted some cultural differences regarding parental attitudes and practices. Secondly, the hierarchical regression showed that physical violence could be partially predicted by the cultural context, childhood abuse, and attitudes in favor of CP. Finally, childhood abuse and parental attitudes mediated the link between the cultural context and parental practices. This study underscores the importance of considering the cultural context when examining parental practices. Moreover, these results provide a better understanding of these types of parental practices in less studied contexts.

## Introduction

Violence is a widespread issue affecting many children (Cuartas et al., 2019; Enzmann & Kammigan, 2018; Hillis et al., 2016; Pinheiro, 2006; Rao & Lux, 2012). Globally, it is estimated that one billion children aged 2 to 17 years have endured physical, sexual, or emotional violence, or neglect in the last 12 months (Hillis et al., 2016), although Article 12 of *the Convention on the Rights of the Child* states that children must be protected from every kind of physical or mental maltreatment. Furthermore, banning corporal punishment (CP) is recommended in every context (Committee on the Rights of the Child, 2006). However, according to the Global Initiative To End All Corporal Punishment Of Children, in 2021, only 62 states in the UN had laws that totally prohibited the use of CP of children (GIEACP, 2021). Furthermore, 1.1 billion caregivers from all over the world deemed CP necessary to discipline children (UNICEF, 2017). Since this practice can have many negative repercussions on a child’s development and later on an adult’s personality and parenting (Cuartas et al., 2021; Durrant & Ensom, 2017; Gershoff & Grogan-Kaylor, 2016; Jaffee & Maikovich-Fong, 2011), the present study aimed to better understand the role played by parent’s childhood experiences of violence and cultural context in the adoption of physically violent parental practices in contexts less documented in the literature, namely Cameroon, Togo, and Switzerland.

### Parental Attitudes and Practices

Physical disciplinary practices are not easy to define (Fréchette & Romano, 2017), but the concept of physical violence toward children can be represented by a continuum of parents’ behaviors going from minor to severe (Clément et al., 2009, 2018; Gershoff, 2002) although the boundary between these two poles is delicate to draw (Coleman et al., 2010). On the one hand, minor physical violence can be defined as all kinds of physical action aiming to correct or control a child’s behavior judged inappropriate by a parent by inflicting pain without injuring (e.g., spank; hit barehanded on the hand, the arm, or the leg; or even pinch). These practices are considered minor when socially accepted. Since this conception depends in particular on social norms (Lokot et al., 2020), there is a great variance between countries in what is considered minor forms of violence. In fact, a panel of experts, convened by the International Society for the Prevention of Child Abuse and Neglect (ISPCAN), discussed the classification of a practice into one category or the other. This resulted in the ICAST, which is an instrument to measure parental behaviors (Runyan et al., 2009). From a legal point of view, laws are currently changing, notably regarding CP. Indeed, in the past few years, many countries, such as France in 2019 (GIEACP, 2021), have adopted a law prohibiting CP. On the other hand, severe physical violence refers to behaviors that are forbidden by law and that can badly injure a child (e.g., choke, punch, kick, hit with a hard object like a belt, or even throw the child on the ground; Clément et al., 2009, 2012). Concerning these two types of practices, several studies have shown that CP increases the risk of severe physical violence (Fréchette et al., 2015; Gershoff & Grogan-Kaylor, 2016; King et al., 2018; Zolotor et al., 2008).

Parents’ beliefs about CP, and in particular attitudes toward it, appear to play an important role in whether or not these practices are adopted (Clément et al., 2012). As defined by Holden (2020), attitudes relate to the negative or positive evaluations of practices. Believing that CP is an effective method refers to a positive attitude toward CP. Research has shown a link between parental attitudes about CP and its use (Ateah & Durrant, 2005; Vittrup et al., 2006; Wang et al., 2018). For instance, in the study of Ateah and Durrant (2005), a positive attitude toward CP was a strong predictor of the use of CP.

Parental attitudes are not the unique factor influencing the use of CP. Parent’s gender, biological aspects, personality, and child characteristics (such as gender) are factors that can influence parenting, and mutual influence may also occur (Bates et al., 2012; Grogan-Kaylor & Otis, 2007; Prinzie et al., 2009; Reijman et al., 2016; Stith et al., 2009). Childhood experiences and contextual factors, such as culture, contribute to forming parental attitudes and practices (Holden, 2020; Rubin & Chung, 2006).

### Intergenerational Transmission of Violent Parental Practices

Intergenerational transmission is one of the most studied factors in the field of physical violence toward children (e.g., Clément et al., 2012; Greene et al., 2020; Kemme et al., 2014; Madigan et al., 2019; McKenzie et al., 2021; Wang et al., 2018). Although the causes of violent parental practices are multifactorial (Savage et al., 2019), experiencing childhood violence remains an important risk factor (Assink et al., 2018). For instance, the study of Clément et al. (2012) highlighted that the risks of using physical violence as a disciplinary method are higher if parents experienced violence during their childhood. In their systematic review, Greene et al. (2020) found that experiencing physical violence or witnessing violence as a child increases the risk of a parent adopting violent practices. Recently, the meta-analytic review of Madigan et al. (2019) examined with a rigorous methodology the intergenerational transmission of child maltreatment and found a modest link concerning the transmission of violence. It seems important to point out that these studies reported research mainly conducted in Western countries.

The relationship between childhood violence and parental practices appears complex. For instance, Clément et al. (2012) found several predictors of violent parental practices in mothers who had experienced childhood violence, such as favorable attitudes toward CP and perceived legitimacy of violence in their childhood. Also, Bower-Russa (2005) identified that the experience of violence during childhood leads to more acceptance of physical disciplinary strategies. More recently, Witt et al. (2021) found out that participants who reported positive attitude toward CP were more inclined to report having experienced CP during their childhood. Finally, several other studies have shown an association between being spanked during childhood and being in favor of CP as a parent (Clément & Chamberland, 2009; Gagné et al., 2007; Simons & Wurtele, 2010).

In sum, understanding the link between childhood experience of parental violence and current disciplinary practices is challenging due to their complex interactions. Other factors, especially culture, have proven to play an important role in the construction of parenting (Bornstein, 2012).

### Influence of Culture on Parental Practices

Taking cultural context into consideration is fundamental to have a better understanding of parenting. According to Bornstein (2012), culture is considered as “the set of distinctive patterns of beliefs and behaviors that are shared by a group of people and that serve to regulate their daily living” (p. 212). These beliefs and behaviors shape the way parents take care of their children. Norms, ideas, and values can differ depending on the culture (Rubin & Chung, 2006), which means that beliefs and practices are, in turn, subject to these variations as well. The influence of culture on parental attitudes can be extremely powerful. Indeed, parents may consciously decide to act on the belief system and behavior model defined by their culture rather than on what they feel about their children’s rearing (Bornstein, 2012; Rubin & Chung, 2006).

When parenting is investigated from a cultural perspective, the two opposed concepts of culture-universal and culture-specific are relevant. First, an attitude or practice is considered universal when it has the same significance and purpose in different cultures (Bornstein, 2012; Rubin & Chung, 2006). Regardless of the cultural context in which parents live, their main concerns will always be how to take care of their children, how to raise them, and how to teach them the way to live in their own culture (Bornstein, 2012). Conversely, when different parental attitudes and practices have different aims in different contexts, they are culturally specific. In a more complex way, different parental attitudes and behaviors may have the same function in different cultures, whereas the same parental cognition and practice may have different purposes in different cultural contexts (Bornstein, 2012; Rubin & Chung, 2006).

A specific parental practice, such as spanking, can be judged to be violent in one culture but less harmful in another one (Bornstein, 2012). Thus, parental practices can vary considerably from one culture to the next. Every culture encourages unique ways of adapting to environmental characteristics and develops parental ethnotheories and traditions that guide children’s rearing. Parents are influenced by a culture-specific set of instructions concerning what is good, accepted, and expected from childcare and parenting (Rubin & Chung, 2006). Besides, in cultural contexts where violence is more tolerated and normative, rates of CP are higher (Lansford & Dodge, 2008).

### Cross-Cultural Differences

Given the intricate links between parenting and culture (Bornstein, 2012; Harkness et al., 2007; Rubin & Chung, 2006), a cross-cultural approach to these subjects appears to be extremely appropriate. In fact, the investigation of various cultures allows a better understanding of the factors involved in parenting since it brings to light those elements that have an influence but are imperceptible when considered from a monocultural point of view (Rubin & Chung, 2006).

In many African societies, CP is socially accepted and is not considered as a form of violence. This is supported by the belief that children have to suffer physically or psychologically to be ready to get ahead in a difficult world (WHO, 2010). Legally, an excess of violence is punished, but some practices are tolerated if their use is deemed beneficial and educational for the child (Menick, 2016). A major issue with this aspect seems to be the definition of a limit between educational practices and maltreatment. From a legal perspective, CP is prohibited in every context in Togo since 2007. It is one of the few countries to have banned it in all settings in sub-Saharan Africa (GIEACP, 2019). In their study conducted in Togo, Dassa et al. (2005) found that parents considered all the suggested methods of punishment (e.g., spanking and slapping) to be normal and legitimate ways to correct their children’s behavior. Their study also showed that three out of four children reported having experienced some forms of maltreatment in the previous year. More recently, as part of UNICEF’s Multiple Indicator Cluster Surveys (MICS) program, Lansford et al. (2020) reported that, in Togo, 35% of their respondents believed that CP is necessary to discipline a child and that 76% of children experienced CP in the last month. Previously, Klevens and Ports (2017) reported that about 11% of Togo’s children aged between 1–14 years old experienced severe physical violence in the past month. A comparative study between France and Togo revealed that Togolese participants experienced more childhood maltreatment than the French ones. Mainly, the prevalence of physical abuse and physical neglect in Togo were twice as high as in France (Kounou et al., 2015). Likewise, in Cameroon, the study of Lansford et al. (2020) revealed that 68% of children experiences CP in the last month and that 43% of the respondents believed that CP is necessary to rear a child. In that country, CP is not prohibited in the family setting and in some care institutions (GIEACP, 2019).

In Switzerland, although CP is considered unlawful, it is not legally prohibited in every context. Indeed, the debate concerning the prohibition of CP in the family setting is currently very strong in Switzerland. It is one of the few countries in Western Europe which has not yet banned CP in all settings. At some levels, CP is still socially accepted. In fact, according to a survey conducted in 2007, 68.1% of the respondents claimed that slapping a child is a legitimate educational method (GIEACP, 2019). Moreover, according to a study conducted by Schmid (2018), it has been estimated that every year, 2 to 3.3% of children in Switzerland are referred to child protection organizations. However, it should be considered that statistical data concerning violence against children show the actual situation only partially since such a topic is still taboo in Switzerland, and also because violence often occurs in a private sphere and hence stays hidden (Schöbi et al., 2018).

### Present Study

Most of the research conducted on the relationships between childhood abuse and parental practices in adulthood has been carried out in Western countries (Serpell & Nsamenang, 2014). Moreover, research is still needed in non-Western countries to collect data concerning violence against children, especially in Sub-Saharan Africa contexts with a colonized past. The Western gaze often considers all of Africa as uniform. However, it seemed important to be able to study countries separately to understand their specificities. In the present study, the focus was on Togo and Cameroon, both of which belong to Sub-Saharan Africa. Thus, this research provides country-specific knowledge. In addition, these contexts are less documented in the literature as shown by the study of Thalmayer et al. (2021). Samples from Africa are under-represented in the field of psychology. Moreover, the focus of this study was also on Switzerland. There have been few studies conducted in this context as well, especially in the French-speaking part of the country. French is also an official language in Togo and Cameroon due to the French colonial past. Although these three contexts share the same language, the norms and laws are different. Finally, it seemed interesting to examine differences in the mechanisms studied in one Westernized country (Switzerland) and two non-Westernized countries (Togo and Cameroon).

To our knowledge, there has been no study pertaining to the links between parental attitudes in favor of CP, the experience of physical abuse during childhood, and physically violent parental practices in such different contexts. The first objective of this study was to document the different forms that physical violence can take and the percentages of attitudes in favor of CP across the three contexts. The second objective aimed, on the one hand at understanding the influence of the cultural context, the childhood physical abuse, and the parental attitudes in favor of CP on the adoption of physically violent parental practices, and on the other one, at investigating whether a mediating effect of the last two variables can be identified between cultural contexts and the adoption of physically violent disciplinary practices.

## Method

### Ethical Procedure

A convenience sample was recruited in the three countries of interest by students in human and social sciences. Ethical approval has been sought from the Vaud Cantonal Ethical Committee. To take part in the study, parents had to be the caregiver of at least one child between 2 and 17 years old. If they had more than one child, they were asked to answer for the youngest one. Moreover, parents had to be over 18 years old and speak French (see Table 1). The participants’ decision to take part in this study was voluntary and in accordance with the ethical norms of the Swiss Society for Psychology. Before starting to fill out the questionnaire, parents had to read the first page indicating that they consented to the study by starting to fill out the questionnaire. They were free to stop whenever they wished and were not remunerated. Above all, participants’ anonymity and data confidentiality were guaranteed. Since the questions could negatively affect participants' psychological well-being, different contacts were suggested for each country to offer them psychological and professional support. Moreover, professional assistance was suggested if needed with children’s education.

**Table 1:** Characteristics for the Three Samples

Characteristics	Cameroon (*n* = 201)		Switzerland (*n* = 147)		Togo (*n* = 199)	
	*n*	%	*n*	%	*n*	%
Parent’s gender: Female	128	63.68	136	92.52	105	52.76
Child’s gender: Female	95	48.22	73	50.00	108	55.39
Geographical region Urban Peri-urban Rural	164 23 10	83.25 11.68 5.80	59 48 40	40.14 32.65 27.21	141 46 8	72.31 23.59 4.10
Education level Primary Secondary Professional Superior	20 50 37 91	10.10 25.25 18.69 45.96	1 4 27 109	0.71 2.84 19.15 77.31	29 39 47 80	14.87 20.00 24.10 41.03
Religion Catholic Protestant Muslim Traditional beliefs Irreligion Other	115 48 10 3 7 17	57.50 24.00 5.00 1.50 3.50 8.50	54 20 5 0 53 7	38.85 14.39 3.60 0 38.13 5.04	84 44 13 5 7 42	43.08 22.56 6.67 2.56 3.59 21.54
Childhood physical abuse above CTQ cut-off ^a^	74	36.82	13	8.84	48	24.12

^a^Parents above the cut-off, which is at 11, were considered to have experienced childhood physical abuse

### Participants

The sample was composed of 547 parents, between 18 and 70 years old (*M* = 36.81, *SD* = 8.80), of whom 178 were male (32.54%) and 369 were females (67.45%). They were recruited in Cameroon (*n* = 201, *M*_*age*_ = 34.45, *SD* = 8.70), Switzerland (*n* = 147, *M*_*age*_ = 38.28, *SD* = 6.92), and Togo (*n* = 199, *M*_*age*_ = 38.11, *SD* = 9.66). Moreover, parents indicated the age of their child for whom they were answering. The mean age was 6.87 (*SD* = 4.19) years for the Cameroonian children, 7.05 (*SD* = 4.46) years for the Swiss children, and 8.20 (*SD* = 4.67) years for the Togolese children (see Table 1).

### Measures

The questionnaire included 121 self-report items. It consisted of three measures, presented below, and 16 socio-demographic questions.

#### Violent Parental Practices

The ISPCAN Child Abuse Screening Tool-Parent version (ICAST-P: Runyan et al., 2009) in French was used to assess various types of disciplinary practices implemented by parents of children under 18 years old over the last year. This questionnaire is composed of 38 items and includes six categories: neglect, sexual abuse and four types of discipline (non-violent, moderate physical, severe physical, and psychological). In the present study, only the moderate (e.g., spanked, hit on buttocks with an object) and severe (e.g., choked, kicked) physical discipline subscales were considered, with respectively 11 and 6 items. The severe physical discipline subscale was used exclusively to document the annual prevalence of this type of practice and its cross-cultural differences. This subscale was not used for other purposes, due to the lack of variability. Concerning items ratings, participants could choose between one of the following answers: *Once or twice* = 1, *3 to 5 times* = 2, *6 to 10 times* = 3, *More than 10 times* = 4, *Not this year* = 5, or *Never* = 6. In this research, to evaluate the prevalence of physically violent parental practices in the three samples, dichotomized variables were used, by differentiating parents who had used a violent practice at least once in the past year from those who had not. The ICAST-P has been recommended for assessing child maltreatment in multicultural contexts. In previous research, Runyan et al. (2009) found a very good internal consistency for both subscales. Cronbach’s alphas were 0.86 for the moderate discipline subscale and 0.81 for the severe one.

#### Parents’ Childhood Experience of Violence

The short form of the Childhood Trauma Questionnaire (CTQ-SF: Bernstein et al., 2003) was used to retrospectively measure the frequency and intensity of adverse experiences during the parent’s childhood. In the present study, the French version was used (Paquette et al., 2004) and only the physical abuse subscale was considered (5 items). The items are rated on a 5-point Likert scale ranging from *Never true* = 1 to *Very often true* = 5. For this subscale, ratings above or equal to 11 denote the presence of physical abuse. This cut-off was used for descriptive purposes only. This allowed comparisons with previous research. However, some precautions must be taken concerning the cultural dimension of the cut-off, which was established in the North American context. Following the approach of Kounou et al. (2015), to better represent Sub-Saharan African realities, the wording of certain terms was changed. In their study, Paquette et al. (2004) found excellent reliability for the physical abuse subscale (α = 0.82).

#### Parental Attitudes in Favor of CP

This scale is made up of six items that were adapted and used by Clément et al. (2009). The first two items measure parents’ general attitudes toward CP. They were adapted from the Adult-Adolescent Parenting Inventory (AAPI: Bavolek, 1984). The third item was from Clément et al. (2009) and concerns the perceived efficacy of CP. The last three items came from the Measure of Parental Violence Towards Children from Fortin and Lachance (1996). They assess parents’ attributions regarding how much they blame their child’s behavior to justify the use of CP. The six items are rated on a 4-point Likert-type scale ranging from *Strongly agree* = 1 to *Strongly disagree* = 4. A total score was calculated by summing the six items reversed, with a higher score indicating more attitudes in favor of CP as an educational method. In their study, Clément et al. (2009) found good internal consistency (α = 0.85).

### Data Analyses

To begin, preliminary analyses were conducted to assess the reliability of the subscales used. Then, descriptive analyses were performed to answer the study’s first objective. For these analyses only, the parental attitudes variable was dichotomized in one group including parents who *disagreed* or *strongly disagreed* with the statements for at least half of the items and the other including the rest of the parents. This allowed us to assess the percentages of parents who had attitudes in favor of CP and to compare the three samples concerning their attitudes. For the second objective, a hierarchical regression analysis was performed to investigate the contribution of five predictors in explaining the variance of physically violent (moderate) parental practices. The socio-demographic variables were entered in the first step, including the context, the parent’s gender, and the child’s gender. To compare, on the one hand, two African countries (Togo and Cameroon) and, on the other hand, a non-Western country to a Western country, Togo was chosen as the reference level. Then, in the second step, childhood physical abuse variable was added and finally, the variable concerning parental attitudes in favor of CP was added. Finally, a multiple mediation was conducted to investigate the role of childhood physical abuse and attitudes in favor of CP on the association between the cultural context and the adoption of physically violent practices.

The statistical analyses were conducted using “RStudio” (version 1.2.5019) and “Jamovi” (version 2.0.0.0) software programs. The significance threshold was 0.05 for all statistical analyses. The multiple imputation method (Broc et al., 2016) was used to replace the missing data (2.11%).

## Results

### Preliminary Results

To assess the reliability of the subscales used, Cronbach’s alphas were calculated. Concerning the ICAST-P (Runyan et al., 2009), in the present study, Cronbach’s alphas was 0.84 for the moderate discipline subscale and 0.71 for the severe one. In addition, Cronbach’s alphas were also calculated for each sample. For the two subscales of the Swiss sample, both values were below 0.70. This was not completely statistically satisfactory but provided interesting information from a cultural perspective. As the ICAST was designed by a professional consensus and the omegas were good (between 0.69 and 0.84 for the moderate subscale and between 0.80 and 0.97 for the severe one, depending on the samples), it has been chosen to use the dimensions proposed by Runyan et al. (2009). Then, the reliability of the physical abuse subscale of the CTQ-SF was excellent (α = 0.81), as was the Cronbach’s alpha of the parental attitude (α = 0.85).

### Parental Practices and Attitudes Across Cultures

Regarding childhood physical abuse, 36.82% of the parents in the Cameroonian sample were above the cut-off, 24.12% of the Togolese parents, and 8.84% of the Swiss parents were also above it. In addition, the prevalence of parents who reported physically violent practices at least once during the previous year was examined. The highest rate of reported moderate violent practices was among the Cameroonian parents at 92.54%, followed by the Togolese parents at 91.46%, and finally by the Swiss parents at 51.70%. Then, 40.30% of the parents from Cameroon, 25.13% of those from Togo, and 6.80% of those from Switzerland mentioned severe physically violent practices at least once. More specifically, across the three contexts, the annual prevalence of some practices was rather similar, whereas some others were different (see Table 2). For instance, regarding similar practices, the hair-pulling rate was between 8.54% and 12.44% for all three countries. Additionally, more parents from Cameroon (30.35% and 32.29%) than from Togo (9.05% and 16.08%) and Switzerland (2.72% and 17.01%) reported kicking and slapping. Some practices seemed more common in African contexts. For example, more Cameroonian (32.84% and 17.41%) and Togolese (30.65% and 19.60%) parents than Swiss parents (3.40% and 4.08%) mentioned hitting on the head with the fist or the back of the hand and beating. Furthermore, some practices, such as spanking and hitting on buttocks with an object, were found to be different in every context: more parents from Cameroon (69.65% and 71.64%) than from Togo (48.74% and 48.24%) and Switzerland (32.65% and 1.36%) reported one of these practices, and more parents from both African countries than from Switzerland mentioned them.

**Table 2:** Frequencies and Chi-Square Results for Physically Violent Practices Across the Three Contexts

Type of violent practices	Cameroon		Switzerland		Togo	
	*n*	%	*n*	%	*n*	%
**Moderate physically violent practices subscale**	**186**	**92.54**	**76**	**51.70**	**182**	**91.46**
Shook child	142	70.65	31	21.10	129	64.82
Hit on buttocks w/ object	144	71.64	2	1.36	96	48.24
Hit elsewhere w/ object	85	42.29	0	0	114	57.29
Twisted ear	121	60.20	20	13.61	70	35.18
Hit on the head w/ fist or the back of the hand	66	32.84	5	3.40	61	30.65
Pulled hair	25	12.44	18	12.25	17	8.54
Chili pepper mouth	8	3.98	5	3.40	7	3.52
Painful kneel/stand	82	40.80	2	1.36	67	33.67
Spanked	140	69.65	48	32.65	97	48.74
Pinched	83	41.29	11	7.48	98	49.25
Slapped	66	32.29	25	17.01	32	16.08
**Severe physically violent practices subscale**	**81**	**40.30**	**10**	**6.80**	**50**	**25.13**
Kicked	61	30.35	4	2.72	18	9.05
Choked	10	4.98	5	3.40	7	3.52
Smothered	6	2.99	6	4.08	4	2.01
Burned	3	1.49	6	4.08	5	2.51
Beaten	35	17.41	6	4.08	39	19.60
Threaten w/ knife or gun	5	2.49	6	4.08	5	2.51

The global scores are in bold

Regarding parental attitudes, the percentages of parents who reported attitudes in favor of CP were also calculated. The highest percentage of attitudes in favor of it was in Cameroon (54.23%), followed by Togo (44.22%), and finally Switzerland (4.76%). More specifically, the percentages for the items 1 and 3 (see Table 3) were rather similar in Togo and Cameroon, but different compared to Switzerland. Concerning the other items, the percentages of parents with attitudes in favor of CP were different in every context, each time with the lowest percentages being for the Swiss parents and the highest, for the Cameroonian parents.

**Table 3:** Frequencies and Chi-Square Results for Attitudes in Favor of CP Across the Three Contexts

Types of attitudes	Cameroon		Switzerland		Togo	
	*n*	%	*n*	%	*n*	%
**Attitudes in favor of CP**	**109**	**54.23**	**7**	**4.76**	**88**	**44.22**
1. There should be a law that allows parents to use force to correct a child	87	43.28	4	2.72	85	42.71
2. Some children need to be slapped to learn how to behave	171	85.08	19	12.93	147	73.87
3. Spanking is an effective method of educating a child	70	34.83	9	6.12	66	33.17
4. It would be acceptable for a parent to hit a child when that child is provocative	127	63.18	13	8.84	102	51.26
5. It would be acceptable for a parent to hit a child when that child is disobedient	132	65.67	10	6.80	114	57.29
6. It would be acceptable for a parent to hit a child when that child is abusive	113	56.22	14	9.52	88	44.22

The global score is in bold. The items are reported in this table as formulated in the questionnaire and freely translated into English. Each line indicates the frequencies and percentages of parents who reported attitude in favor of the statement

### Factors Influencing Parental Practices

Concerning the hierarchical regression analyses, the socio-demographic variables explained 27.3% of the moderate violent practices (MVP) variance (*F*(4, 533) = 50.0, *p* < 0.001). The influence of childhood physical abuse, entered for the second step of the model, was statistically significant (*p* < 0.001) and explained an additional 3.8% of the variance of the MVP. The addition of the attitudes in favor of CP in the third step explained an additional 3.5% of the variance of the MVP (p < 0.001). The overall model is significant (*F*(6, 531) = 46.9, *p* < 0.001) and explain 34.7% of MVP variance. As depicted in Table 4, all the predictors included in the model emerged as statistically significant predictors of MVP. However, the cultural context had a significant effect only when comparing Switzerland and Togo (*ß* = -0.67, *p* < 0.001). Then, female parents used less MVP than male parents (*ß* = -0.19, *p* < 0.05) and boys experienced more MVP than girls (*ß* = 0.20, *p* < 0.01). Parents who had experienced more physical abuse in childhood reported more MVP (*ß* = 0.20, *p* < 0.001) and parents who reported more attitudes in favor of CP as well (*ß* = 0.25, *p* < 0.001).

**Table 4:** Hierarchical Regression Analysis Results Predicting Moderate Physically Violent Parental Practices

Predicting factors	*ß*	*t*	*p*	Adjusted* R*^*2*^	*R*^*2*^* change*
**Step 1**				.27	
Cultural context Cameroon – Togo Switzerland – Togo					
	.07	0.80	.42		
	-.67	-5.82	< .001		
Parent’s gender Male – Female	-.19	-2.39	< .05		
Child’s gender Male – Female	.20	2.81	< .01		
**Step 2**				.31	.04
Childhood physical abuse	.20	5.31	< .001		
**Step 3**				.34	.04
Attitudes in favor of CP	.25	5.37	< .001		

For the parental attitudes in favor of CP variable, a higher score indicates more attitude in favor of CP

* p < .05; ** p < .01; *** p < .001

**Fig. 1:**
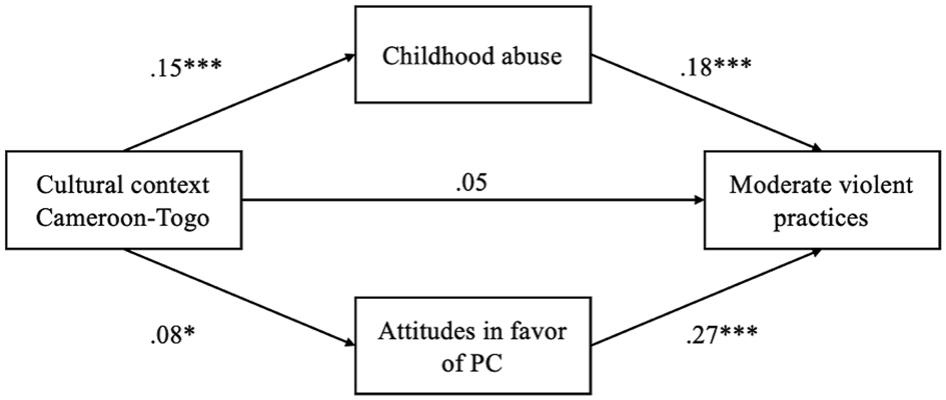
Mediation of childhood experience of violence and parental attitudes in favor of PC on the link between the cultural context (Cameroon-Togo) and physically violent practices. Values indicate standardized coefficients (*β*), *p < .05, ***p < .001

**Fig. 2:**
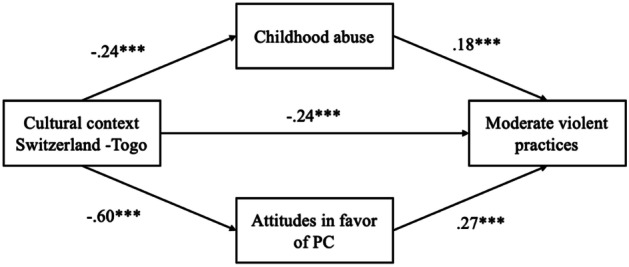
Mediation of childhood experience of violence and parental attitudes in favor of PC on the link between the cultural context (Switzerland-Togo) and physically violent practices. Values indicate standardized coefficients (*β*), *p < .05, ***p < .001

Finally, the multiple mediation analysis explored the mediating role of childhood physical abuse and parental attitudes in favor of CP on the link between the cultural context and physically violent practices. First, the direct effect was not significant when comparing Cameroon and Togo (*ß* = 0.05, *p* = 0.18, 95% CI [-0.48, 2.58]; see Fig. 1) but was when comparing Switzerland and Togo (*ß* = -0.24, *p* < 0.001, 95% CI [-7.15, -3.10]; see Fig. 2). Then, results showed that the indirect effect through the experience of abuse during childhood was significant when comparing Cameroon and Togo (*ß* = 0.03, *p* < 0.01, 95% CI [0.16, 0.92]) and when comparing Switzerland and Togo (*ß* = -0.04, *p* < 0.001, 95% CI [-1.43, -0.42]). The indirect effect through the attitudes in favor of CP was also significant in both cases (Cameroon – Togo:* ß* = 0.02, *p* < 0.05, 95% CI [0.03, 0.83]; Switzerland – Togo: *ß* = -0.16, *p* < 0.001, 95% CI [-4.59, -2.19]).

## Discussion

The first purpose of this study was to document the different forms of parents’ physically violent practices across cultures. The results indicated that physically violent parental practices and attitudes in favor of CP manifest themselves differently according to the three cultural contexts. The second aim was to better understand the influence of the cultural context, the childhood physical abuse, and parental attitudes in favor of CP on physically violent parental practices in Togo, Cameroon, and Switzerland. The findings showed that violent physical practices could be partially predicted by the cultural context, childhood physical abuse, attitudes in favor of CP, child’s gender, and parent’s gender. Moreover, childhood abuse and parental attitudes turned out to play a mediator role on the link between the cultural context and the adoption of physically violent practices.

### Influence of the Cultural Context

In this study, the cultural context was found to play a role in the disclosure of physically violent parental practices. The percentages of any forms of moderate violence reported by the parents were high in Cameroon and Togo, at more than 90% for both countries, which is slightly higher than the results of Dassa et al. (2005) and Lansford et al. (2020). However, the practices evaluated in these studies were slightly different from those evaluated in our study. Our results highlighted the tolerance and acceptance of all these types of violence in daily education. This is consistent with the study of Lansford and Dodge (2008) which found that rates of CP are higher in contexts where violence is normative. In Switzerland, the rate was more than 50%, which is quite high. Even though violence against children is often socially disapproved, Swiss parents still adopt these practices. This may be due to the laws (Zolotor & Puzia, 2010), which still accept CP in the family setting. However, our results mainly indicated cross-cultural differences. Even if every culture had some violent practices, these practices varied in their forms. In Togo and Cameroon, beating seemed to be rather common. This practice is widely used in Sub-Saharan Africa and seems to be culturally specific to these contexts (Dassa et al., 2005). Concerning hair-pulling in these two contexts, this practice was one of the least used. As suggested by Meinck et al. (2018), this could be because, in these contexts, children have short hair. More broadly, severe violent practices were more prevalent in Cameroon than in Togo, which is consistent with the study of Klevens and Ports (2017). These results may reflect, firstly, the laws in Togo prohibiting CP. Secondly, they may reflect the work of NGOs, notably “Plan International—Togo,” which aims to raise awareness of the deleterious effects of CP through media. Concerning Switzerland, spanking, slapping, shaking, and twisting the ears were the most prevalent violent practices. Indeed, the use of spanking is still debated with its supporters and opponents and remains tolerated in the family setting. In addition, the results showed that the use of any object in this context was not a common practice. These practices in Switzerland, as well as the use of chili in all contexts, do not seem to correspond to common and accepted practices in these countries. By focusing on less studied contexts, this research highlighted the differences between a universal conceptualization of a phenomenon and how it manifests in each context.

Furthermore, our results showed that the cultural context seemed to also influence parental attitudes in favor of CP, which supports Bornstein (2012) and Rubin and Chung (2006) studies. In particular, Cameroonian and Togolese parents in this study displayed more attitudes in favor of CP, which reflects the tolerance of CP that can be found in their respective cultures despite international treaties. For their part, Swiss parents seemed to have less attitudes in favor of it. This is in line with their culture which is socially less tolerant toward harsh discipline in parenting.

### Determinants of Violent Physical Practices

The results of the present study showed that experiencing physical abuse during childhood is associated with the adoption of physically violent practices. This is consistent with the meta-analysis of Assink et al. (2018) which found that childhood abuse is an important risk factor. Moreover, in the present research, the experience of abuse turned out to mediate the association between the cultural context and the adoption of violent practices. The results suggested that living in a cultural context that tolerates violent practices would increase the risk that parents experience physical abuse as children and thus increase the risk that they adopt these same practices with their children. It is therefore important to intervene at the level of law and universal prevention to reduce the violence experienced by the next generations.

The experience of physical abuse during childhood and the context are not the only factors influencing parenting. Indeed, our results showed that parents’ attitudes in favor of CP influence parenting. Parents who had more attitudes in favor of CP were more likely to report adopting physically violent practices toward their children. This result is consistent with those of Clément et al. (2012) and Ateah and Durrant (2005), who stated that parental attitudes are a predictor of violent parental practices. More generally, the results confirmed that these attitudes and beliefs can shape parents’ behaviors (Rubin & Chung, 2006). In addition, parental attitudes mediated the relation between the cultural context and the adoption of violent practices, meaning that a culture which is more tolerant of violent parental practices would imply that a parent would have more attitudes in favor of CP and thus would be more likely to adopt violent practices.

Besides, the parent’s gender appeared to play a role in the adoption of physically violent practices, as well as the child’s gender. Being a male parent was related to using more likely physical violence, which is not completely in line with other findings. For instance, the study of Lansford et al. (2010) across nine countries found that, generally, mothers were more likely to use CP than fathers. Besides, differences between fathers and mothers in the adoption of physically violent practices came out in the literature. Sometimes, the fathers used more physical violence with their sons (McKee et al., 2007), while at other times, the mothers used more physical violence with their daughters (Cui et al., 2016). Concerning children, in the present study, being a male child was associated with experiencing more CP. Mehlhausen-Hassoen (2021) also found out that sons experienced more CP than daughters, as well as Lansford et al. (2010) in their study.

### Practical implications and Future Research

As described above, since attitudes toward CP were associated with the adoption of physically violent practices, a modification of these attitudes could be an effective way to prevent the use of physical violence, as Holden et al. (2014) did in their intervention. In addition, it would be important to understand the factors that may lead to favorable attitudes toward violent parental practices. Gagné et al. (2007) showed that parents who are less aware of the consequences of CP are more likely to approve spanking as an educational method. Likewise, Clément and Chamberland (2009) demonstrated that a mother’s sensitivity toward these consequences is an important predictor of maternal attitudes about these methods. As a consequence, prevention programs should aim to raise awareness about the risks of physical violence toward children (Pinheiro, 2006). However, as our findings indicated different links in the three cultural contexts, it seems important to have culturally specific preventive approaches. Violent parenting prevention programs that have proven effective generally include a model of understanding based on social learning theory (e.g., Forgatch & Patterson, 2010). This model helps parents to understand why violence does not work (can worsen the child's behavior, increases parental guilt, risks of injury, etc.). Some selective child maltreatment prevention programs (e.g., Wiggins et al., 2009) go further by specifically targeting parents’ unrealistic expectations of a child's behavior and parents’ causal attributions that can trigger parents’ violent behavior (Azar & Weinzierl, 2005). It would be interesting to investigate if these programs may be suitable also in countries like Togo or Cameroon and help change parental attitudes toward CP. Disagreement concerning the use of violent practices in children’s education is a common feature in parents who break the cycle of violence transmission across generations (Clément et al., 2012).

Future studies could develop more culturally adapted scales to measure violent parental practices, as the conceptualization of these can vary from one context to another. In addition, the conceptualization of violence against children is based on predominantly Western literature. Therefore, it might be interesting to study these different conceptualizations in these contexts through qualitative interviews. Then, future research could include mixed methods, such as observation tasks, to bring out elements that are explored by means other than questionnaires. This could continue with examinations of the needs of parents in these countries, as well as the knowledge and skills of professionals, which is an important step in the implementation of prevention programs in low- and middle-income countries (Brodard & Naudin, 2021). Indeed, this would contribute to the assessment of these countries’ readiness to implement child maltreatment prevention programs (Mikton et al., 2013).

### Limitations

The present study has several limitations. The questionnaires were filled out by the parents themselves. Therefore, the figures reported above are based on their perceptions and memories, and not necessarily a reflection of reality. Due to the self-reported style of the questionnaire, the presence of a social desirability bias may be a considerable limitation of this study (Krumpal, 2013). In fact, despite the guarantee of participants’ anonymity and data confidentiality, participants may not have answered completely honestly due to the sensitivity of a topic like violence against children. Moreover, the impact of the culture on the style of response is another bias to take into consideration (see Udayar & Antonietti, 2021). Besides, this study only studied culture at a certain level, but many other aspects should be considered for a complete vision of what culture includes. Also, as mentioned previously, given the lack of research in these countries, the scientific gaze on the conceptualization of violence against children is often Western. This may have influenced the way this violence was conceptualized in this study, even though the research team was multicultural. Finally, the convenience sample in the present study does not allow the results to be generalized to an entire population. However, for instance, in Cameroon, this seems to be a difficult task because of the great cultural diversity existing in this context (Mfewou, 2018). Therefore, the purpose of this study was more to describe the relations between the variables examined in these understudied contexts rather than having a strict representation of these contexts.

## Conclusion

In light of current concerns about violence against children and its negative consequences, this study was focused on physically violent parental practices. Given the cultural differences in parenting, a cross-cultural perspective seemed relevant. Since the results showed that culture influences parental attitudes toward CP and physically violent practices, this study highlighted the importance of considering cultural background when examining parental practices. Although violent parental practices in Sub-Saharan African contexts have rarely been investigated, these findings could provide a better understanding of these types of parental practices notably for parents from Togo and Cameroun who move to the West. Moreover, the focus on Togo and Cameroon in this study also helped to differentiate parental practices in these two contexts. However, research in this field is still needed, notably to understand how maltreatment is conceptualized in these contexts. In addition, this research highlighted the mediating role of the experience of violence during childhood and the parental attitudes in favor of CP, providing new findings regarding mechanisms underlying intergenerational transmission in the contexts studied. To conclude, further investigations should be carried out to identify factors involved in violent parenting, to help break the cycle of violence.

## Data Availability

The data that support the findings of this study are available from the corresponding author [C.N.], upon reasonable request.
